# Use of integrated clinical trial protocols – A survey in early medicines development 

**DOI:** 10.5414/CP203206

**Published:** 2018-02-16

**Authors:** Katharina Erb-Zohar, Hildegard Sourgens, Kerstin Breithaupt-Groegler

**Affiliations:** Association for Applied Human Pharmacology (AGAH e.V.) on behalf of the AGAH Working Group “Diskussionsforum Zukunftskonzepte der frühen klinischen Prüfung”, Hamburg, Germany

**Keywords:** integrated protocol, combined protocol, umbrella protocol, clinical trial, early medicines development, survey

## Abstract

Purpose: To collect information on the use of integrated protocols in early clinical medicines development. Materials and methods: The questionnaire was mailed in fall 2014 to members of human pharmacology societies in Europe for anonymous responses via the online tool SurveyMonkey^®^. Results: 97 respondents reported on 164 integrated protocols overall. In general, integrated protocols comprised 2 or 3 trial elements. One third of integrated protocols involved patients. The most frequent trial elements were single dose, multiple dose, and food effect. Drug-drug interaction, age, gender, and relative/absolute bioavailability were less common elements. Ethnic bridging and mass balance were mentioned in single cases. Out of the entire spectrum of reported trial element combinations, single (ascending) dose plus multiple (ascending) dose was most frequent (90/164 protocols, 55%); 84% of integrated protocols used adaptive elements. 29%, 17%, and 8% of integrated protocols required 1, 2, or 3 substantial amendments, respectively. Based on 118 protocols, competent authority approval was granted to 100, deficiency letters were issued 15 times and approval was refused in 3 cases. Conclusion: The use of integrated protocols is common practice in early medicines development. Most often single ascending dose and multiple ascending dose were the trial elements combined in one integrated protocol. Perceived main advantages were gain in time and reduced costs. Perceived main disadvantage was increased complexity.

## Introduction 

A series of workshops related to early phase clinical development has been hosted by the scientific society “Association for Applied Human Pharmacology” (AGAH e.V.) in Germany since 2012. In 2014, members from the pharmaceutical industry, contract research organizations, academia, ethics committees, and the German competent authority evaluated the usability of integrated protocols; see http://www.agah.eu/infothek/workshops/archiv/agah-diskussionsforum-zukunftskonzepte-der-fruehen-klinischen-pruefung-1.html. 

A survey was conducted to collect information on the frequency of use and the design features of integrated protocols across European countries. In addition, the number of substantial amendments related to these protocols was inquired as well as the perceived advantages/disadvantages. 

## Materials and methods 

The survey addressed specialists working in early phase medicines development in Germany, Belgium, France, and the United Kingdom. 

The following definitions were applied:

- Integrated protocol (synonyms: combined protocol, umbrella protocol): clinical trial protocol that combines more than one trial element, such as single ascending dose plus food effect or single ascending dose plus multiple (ascending) dose
- Adaptive elements: the protocol includes elements of flexibility, e.g. dose, sample size, sampling time-points, measurements, overlap of trial elements

The questionnaire (Appendix 1) comprised 25 questions for anonymous responses by means of the online tool SurveyMonkey^®^. Addressees in Germany were AGAH members, delegates of the VfA (“Verband forschender Arzneimittelhersteller”), BPI (“Bundesverband der pharmazeutischen Industrie”), BAH (Bundesverband der Arzneimittel-Hersteller), DGPharMed (Deutsche Gesellschaft für Pharmazeutische Medizin’); in Belgium members of the Belgian Association of Phase I units (BAPU); in France members of Club Phase I; and in the United Kingdom members of the Association for Human Pharmacology in the Pharmaceutical Industry (AHPPI). E-mailings were on July 30, 2014 and September 23, 2014. The survey was closed on October 16, 2014. 

The questions covered the following topics (see questions (Q) provided in Appendix 1):

- Characteristics of survey participants (Q1 – Q3)
- Number and type of trial elements combined in one protocol (Q4 – Q6)
- Trial population recruited for integrated protocols (Q7)
- Use of adaptive design elements (Q8 – Q16)
- Number of substantial amendments (Q17)
- Competent authorities (CA) involved in clinical trial applications (CTAs) and their feedback (Q20, Q21)
- Advantages vs. disadvantages (Q22 – Q25)

It cannot be excluded that different participants belonging to the same organization referred to the same trial protocol(s). In consequence, a qualitative rather than quantitative interpretation of results is indicated. Not all survey participants answered all questions. For the respective questions, the total number of responses is given. 

Q6 asked for trial elements that were combined in the integrated protocol. Up to 3 representative examples could be given. The total of all reported combinations of trial elements, number of combined elements and individual combinations were counted and tabulated. The data was evaluated descriptively. 

## Results 

### Survey participants 

97 of the addressees answered the questionnaire. The majority came from the pharmaceutical industry (37) and contract research organizations (31), but also from academia (13), independent consultants (10), biotech, or others (10). 

Participants were from Germany (61), Belgium (18), United Kingdom (8), and other countries (Switzerland 4, Eastern Europe 2, The Netherlands 1, France 1, USA 1, Asia 1). 

The size of institutions or companies (86 answers) was from < 10 to < 100 employees for ~ 1/3 and > 100 to > 1,000 for ~ 2/3 of survey participants. 

### Use of integrated protocols 

A total of 171 protocols were evaluated; 164 were integrated protocols, 7 protocols used one trial element. 75 of 96 survey participants (78%) confirmed having conducted integrated protocols in early medicines development. Out of these, 64 participants specified how many integrated trials they conducted within the last 5 years: 39% of participants had conducted more than 10 trials, 27% 3 – 5 trials, 20% 1 – 3 trials, and 14% 5 – 10 trials. 

Moreover, 64 participants specified which individual trial element combinations they had used in up to 3 integrated protocols. The most frequent trial elements were single (ascending) dose, multiple (ascending) dose, and food effect (Table 1). Drug-drug interaction, age, gender, and relative/absolute bioavailability were less common elements (Table 2). Ethnic bridging and mass balance were mentioned in single cases. 

### Trial population (64 respondents, 168 protocols) 

Most integrated protocols involved healthy subjects exclusively (70%), followed by healthy subjects plus patients (19%), and patients only (11%). 

### Use of adaptive elements (51 respondents, 128 protocols) 

84% of respondents used adaptive elements. These comprised dose (increments), timing of trial elements (e.g., parallel or independent conduct of parts), timing and sampling of pharmacokinetics and safety laboratory as well as assessment schedules, and sample size. The majority of respondents (82%) reported no issues with the use of adaptive elements. However, complex integrated protocols required repeated amendments. Moreover, problems with drug supply and recruiting had been observed. 

### Number of substantial amendments (50 respondents, 132 protocols)

29%, 17%, and 8% of integrated protocols required 1, 2, or 3 substantial amendments, respectively. 42% of integrated protocols did not require any substantial amendment; 4 or more substantial amendments were reported for less than 5% of integrated protocols. 

### Competent authorities involved and feedback received (44 respondents, 123 protocols)

**Figure 1.**
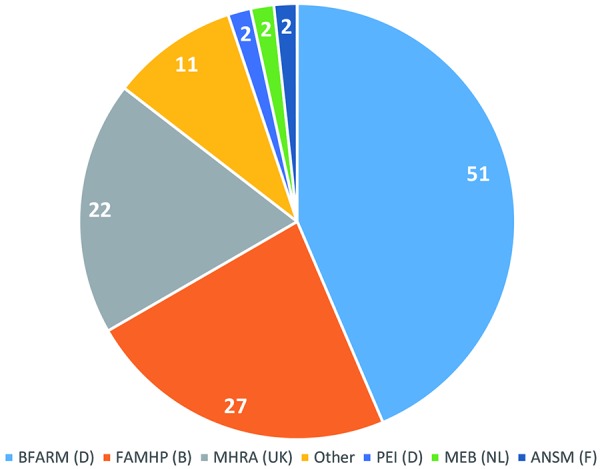
Distribution of submitted clinical trial applications across competent authorities (based on 123 integrated protocols, given are numbers of protocols submitted to authority). BfArM = Federal Institute for Drugs and Medical Devices, Germany (D); famhp = Federal Agency for Medicines and Health Products, Belgium (B); MHRA = Medicines and Healthcare Products Regulatory Agency, United Kingdom (UK); PEI = Paul Ehrlich Institute; Germany (D); MEB = Medicines Evaluation Board, The Netherlands (NL); ansm = National Agency for the Safety of Medicine and Health Products, France (F); Other: Irish Medicines Board, Japanese Ministry of Health and Welfare, Hungarian Authority.

**Figure 2.**
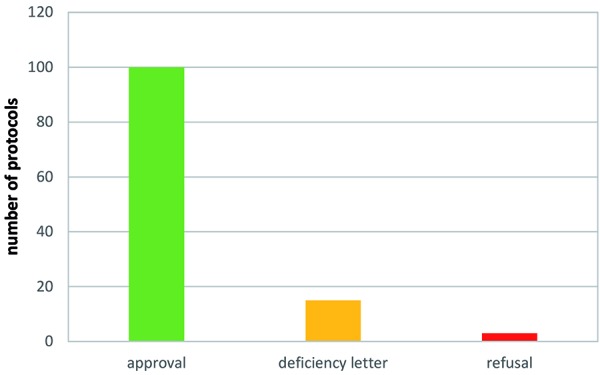
Competent authority approval, deficiency letter, and refusal of integrated protocols (based on 118 protocols).

Competent authorities to whom clinical trial applications with integrated protocols were submitted are detailed in Figure 1, while outcome of authority evaluation is given in Figure 2. Eleven deficiency letters concerned protocols in healthy subjects and two concerned protocols in healthy subjects plus patients. 

Specified deficiencies were:

- Insufficient definition of in/exclusion criteria
- Lack of or insufficient stopping rules
- Lack of interim safety and pharmacokinetic data for the dose escalation part before starting subsequent parts of the trial
- Imprecise and unjustified dose selection
- Request to provide multiples of exposure/safety margins based on non-clinical data for the dose escalation parts

### Perceived advantages of integrated protocols

- Saving of time (1 – 6 months vs. single protocols), resources and total costs
- One regulatory step
- Learning from emerging data
- Reaching effective dose in patients earlier

### Perceived disadvantages of integrated protocols

- Complexity
- Requirement for substantial amendments
- Time-consuming preparation
- Difficult handling with regard to conduct, data management, analysis, reporting

## Discussion 

This survey on the use of integrated protocols in early medicines development, to the best of our knowledge, is the first to provide hands-on data on present practice across Europe. Integrated protocols are used in phase I trials with healthy subjects at least since 2004 in Germany [1] and at least since 2006 in the UK [2]. 

The main outcome of this AGAH survey demonstrates the wide-spread use of this approach in early clinical development. The update of the “Guideline on strategies to identify and mitigate risks for first-in-human and early clinical trials with investigational medicinal products” (EMEA/CHMP/SWP/28367/07 Rev. 1, coming into effect on February 1, 2018 [3]) reflects the “increasing practice to perform first-in-human and early phase clinical trials with integrated protocols that combine a number of different study parts” and details requirements for safe and state-of-the-art practices. 

According to the survey results, integrated protocols often comprised 2 or 3 trial elements. Combining single ascending and multiple ascending dose parts in one protocol was very common. Likewise, assessing food effects as an additional trial element was stated frequently. Less frequently, integrated protocols comprised up to 7 trial elements. Survey participants perceived time and cost saving, learning from emerging data as well as one regulatory step only as advantages of integrated protocols. In a retrospective analysis of 29 clinical trials with adaptive elements, thereof 14 integrated protocols, conducted at a single center in the UK [2], time saving (56 days on average) was achieved by using adaptive elements. Most of the participants of this AGAH survey used adaptive elements in their integrated protocols. Interestingly, according to an analysis of clinical trial applications concerning early medicine development submitted to the Federal Institute for Drugs and Medicinal Devices (BfArM), the authorization procedures with integrated protocols took significantly longer until initial authorization (58 vs. 53 days) required more substantial amendments (1.9 vs. 1.2 amendments per clinical trial application) and the approval of the entirety of amendments took longer to process as compared to subsequent standard protocols (22 vs. 14 days). However, time and/or cost may be saved (no further details provided) with integrated protocols during medicines development because overall less clinical trial applications and less clinical trials are needed than with non-integrated protocols [1]. 

In Germany, time savings can also be achieved using accelerated approvals for a series of clinical trial applications of clinical trial protocols that build upon each other [4, 5] 

Perceived disadvantages of integrated protocols were trial complexity including protocol preparation, logistics, informed consent, trial conduct, data management, analysis, and reporting. Trial preparation was perceived as time-consuming. Complex protocols were reported to increase the number of substantial amendments. 

Adaptive elements (like increments of dose, timing of trial elements, timing of PK, PD and safety assessments, or sample size) were essential design features of integrated protocols as confirmed by the majority of survey participants. As part of the general risk minimization process, adaptive elements require the upfront definition of limits in the trial protocol within which they may be applied (reviewed in [6]). 

Limitations of the reported survey are that the entirety of participants is not a representative sample of all parties conducting clinical trials in Europe, and participants from the same organization may have referred to the same trials. Strengths of the reported survey are the variety of topics covered and the derived information on, e.g., combinations of trial and adaptive elements which reflects current common practice. 

During the workshop held in 2014, there was agreement that integrated protocols and their clinical trial elements must be scientifically sound, state-of-the-art and must not increase the risk for clinical trial participants. For the combination of single ascending dose and multiple ascending dose, a safe corridor of exposure, technical prerequisites (rapid availability of pharmacokinetic data), predefined decision trees and stopping rules were discussed. Good practices in the planning and conduct of clinical trials with integrated protocols in early medicines development comprise thorough risk assessments in the planning phase, appropriate risk minimization measures including clearly predefined algorithms for decision-making and stopping rules that unambiguously can be applied during trial conduct [7, 8]. 

## Conclusion 

Among the participants of this AGAH survey, the use of integrated protocols in early medicines development is common practice. Most often, single ascending dose and multiple ascending dose are among the trial elements combined in one integrated protocol. Main perceived advantages are a gain in time and reduced costs. The main perceived disadvantage is increased complexity. 

## Acknowledgment 

We thank Ulrike Lorch for her contribution to the concept and the content of the survey and Sam Welgemoed for her technical support (Richmond Pharmacology, London, and St. George’s University London, UK). We thank Andreas Kovar for reviewing the questionnaire (Sanofi-Aventis Deutschland GmbH, Frankfurt, Germany). We thank Jens Rengelshausen for reviewing the questionnaire and minuting the survey discussions (Grünenthal GmbH, Aachen, Germany). We thank all participants of the survey for their contributions. 

## Funding 

The Association of Applied Human Pharmacology (AGAH e.V., Hamburg, Germany) is a learned society and funded open access. 

## Conflict of interest 

None. 

## Appendix 1

[Table Appendix1]

**Table 1.** Frequent combinations in integrated protocols (based on 164 protocols).

Number of trial elements within integrated protocols	Combined trial elements	Number
87 protocols with 2 trial elements	SD+MD	34
	SD+food	20
	SD+abs/rel BA	6
	Food+abs/rel BA	5
	MD+food	5
	Gender+abs/rel BA	3
	Abs/rel BA+mass bal	2
	MD+DDI	2
	Various^a^	10
56 protocols with 3 trial elements	SD+MD+food	20
	SD+MD+abs/rel BA	5
	SD+MD+DDI	5
	SD+MD+gender	3
	SD+food+abs/rel BA	3
	SD+food+gender	3
	SD+MD+ethn br	2
	MD+age+gender	2
	MD+DDI+age	2
	SD+MD+age	2
	SD+MD+ethn br	2
	Various^b^	7

SD = single ascending dose; MD = multiple ascending dose; food = drug-food interaction; DDI = drug-drug interaction; abs/rel BA = absolute/relative bioavailability; mass bal = mass balance; ethn br = ethnic bridging. ^a^age+abs/rel BA (1), age+gender (1), food+age (1), food+DDI (1), food+gender (1), MD+abs/rel BA (1), MD+age (1), SD+DDI (1), SD+ethn br (1), SD+gender (1). ^b^Age+gender+ethn br (1), food+age+gender (1), MD+ethn br+abs/rel BA (1), MD+food+DDI (1), SD+age+gender (1), SD+food+ethn br (1), SD+food+PET occupancy (1).

**Table 2.** Less frequent combinations in integrated protocols (based on 164 protocols).

Number of trial elements within integrated protocols	Combined trial elements	Number
12 protocols with 4 trial elements	SD+MD+age+gender	3
	SD+MD+food+DDI	2
	MD+food+gender+abs/rel BA	1
	SD+food+age+gender	1
	SD+food+gender+abs/rel BA	1
	SD+MD+food+abs/rel BA	1
	SD+MD+food+age	1
	SD+MD+food+ethn br	1
	SD+MD+food+gender	1
7 protocols with 5 trial elements	SD+food+age+gender+abs/rel BA	1
	SD+MD+DDI+age+gender	1
	SD+MD+food+age+abs/rel BA	1
	SD+MD+food+age+gender	1
	SD+MD+food+DDI+age	1
	SD+MD+food+DDI+gender	1
	SD+MD+food+gender+abs/rel BA	1
A total of 2 protocols with ≥ 6 trial elements	SD+MD+food+age+gender+ethn br	1
	SD+MD+food+DDI+age+gender+ethn br	1

SD = single ascending dose; MD = multiple ascending dose; food = drug-food interaction; DDI = drug-drug interaction; abs/rel BA = absolute/relative bioavailability; ethn br = ethnic bridging.

**Appendix 1. Appendix1:** Q: Question, below each question the options that could be selected as answer are given.

Q1	Please select from the list below the option that best describes you or your affiliation: Pharmaceutical industry, Biotech, CRO, University, Consultant, Other
Q2	What is the size of your affiliation: < 10, < 100, < 1,000, > 1,000 employees
Q3	In which country are you based? Belgium, France, Germany, United Kingdom, Other (please specify)
Q4	Have you or your organization ever conducted a clinical trial in early drug development with combined protocols, such as single dose escalation + food study? *Combined protocol = umbrella protocol: protocol that combines more than one study element, such as single ascending dose plus food study or single ascending dose plus multiple dose * Yes, No
Q5	If yes, how many combined protocols did you or your organization conduct within the last 5 years? 1 – 3, 3 – 5, 5 – 10, > 10
Q6	Please give 1 to 3 representative examples (multiple options can be selected here) SD, MD, food, DDI, age, gender, ethnic bridging, absolute and/or relative bioavailability, mass balance
Q7	In which population did you conduct these clinical trials with combined protocols? Healthy subjects, patients, healthy subjects+patients
Q8	Did you use adaptive elements in these combined protocols? *Adaptive elements = the protocol includes elements of flexibility e.g. of dose, sample size, sampling time-points, measurements, or allows overlap of study parts * Yes, No
Q9	If yes: Which? (multiple options can be selected here) Flexibility of dose, sampling and measurements, sample size; parallel or independent conduct of study parts
Q10	Were there any issues with the adaptive elements used in these combined protocols? Yes, No
Q11	If yes, please state the issues
Q12	What happens if adaptive elements are used and changes are made?
Q13	Does this need to be notified to the competent authority and/or the ethics committee? Yes, No
Q14	If notification is required, do you consider this beneficial? Yes, No
Q15	When changes by the use of adaptive elements are made is approval from the competent authority and/or the ethics committee required before these changes can be implemented into the study? Yes, No
Q16	If approval is required, please give an estimate (weeks) how long this would take
Q17	How many substantial amendments did you submit for these trials with combined protocols? None, 1, 2, 3, 4, > 5, > 10
Q18	If you compare the duration of studies within a combined protocol with the duration of the sum of separate protocols is there any difference? Yes, No
Q19	If yes, which (estimate)? (multiple options can be selected here) No time saving, combined protocol took longer than individual studies, time saving of avoiding substantial amendment(s), time saving of avoiding standard regulatory submission, time saving of ethics review times for initial submissions; (for duration differences estimate in months)
Q20	To which competent authorities did you submit a clinical trial application with a combined protocol? famhp (Federal Agency for Medicines and Health Products, Belgium); ansm (National Agency for the Safety of Medicine and Health Products, France); BfArM (Federal Institute for Drugs and Medical Devices, Germany); PEI (Paul Ehrlich Institute, Germany); Medicines Evaluation Board (Netherlands); Healthcare Inspectorate (Netherlands); MHRA (Medicines and Healthcare Products Regulatory Agency, UK); Other (Please specify)
Q21	Which feedback did you receive from the competent authority to your clinical trial application with a combined protocol? Approval, refusal, deficiency letter; specify main deficiencies
Q22	What are advantages of combined protocols in your opinion?
Q23	What are disadvantages of combined protocols in your opinion?
Q24	Do you have any comments on combined protocols? Please specify
Q25	Would you like to add anything else? Please specify
